# Immunotherapy against Prion Disease

**DOI:** 10.3390/pathogens9030216

**Published:** 2020-03-14

**Authors:** Yue Ma, Jiyan Ma

**Affiliations:** Center for Neurodegenerative Science, Van Andel Institute, 333 Bostwick Avenue N.E., Grand Rapids, MI 49503, USA; yue.ma@vai.org

**Keywords:** prion disease, PrP, immunotherapy, vaccine, antibody

## Abstract

The term “prion disease” encompasses a group of neurodegenerative diseases affecting both humans and animals. Currently, there is no effective therapy and all forms of prion disease are invariably fatal. Because of (a) the outbreak of bovine spongiform encephalopathy in cattle and variant Creutzfeldt–Jakob disease in humans; (b) the heated debate about the prion hypothesis; and (c) the availability of a natural prion disease in rodents, the understanding of the pathogenic process in prion disease is much more advanced compared to that of other neurodegenerative disorders, which inspired many attempts to develop therapeutic strategies against these fatal diseases. In this review, we focus on immunotherapy against prion disease. We explain our rationale for immunotherapy as a plausible therapeutic choice, review previous trials using either active or passive immunization, and discuss potential strategies for overcoming the hurdles in developing a successful immunotherapy. We propose that immunotherapy is a plausible and practical therapeutic strategy and advocate more studies in this area to develop effective measures to control and treat these devastating disorders.

## 1. Background

Prion diseases, also known as transmissible spongiform encephalopathies (TSEs), are a group of fatal neurodegenerative disorders affecting both humans and animals [1,2]. Prion disease can be sporadic, genetic, or acquired (infectious). In humans, most cases of prion diseases are sporadic, including sporadic Creutzfeldt–Jakob disease (CJD) and sporadic fatal insomnia. About 5–15% of prion disease cases are genetic (dominantly inherited), including familial CJD, fatal familial insomnia (FFI), and Gerstmann–Sträussler–Scheinker syndrome (GSS). Less than 5% are acquired cases, which includes Kuru, iatrogenic CJD, and variant CJD [3]. The well-publicized vCJD is believed to result from the consumption of beef products derived from bovine spongiform encephalopathy (BSE) affected cattle [4], illustrating the zoonotic potential of animal prion disease. Besides BSE, animal prion diseases also include chronic wasting disease (CWD) in cervids and scrapie in sheep and goats [4]. 

The infectious agent (the prion) of prion disease is known as PrP^Sc^. It is the misfolded form of the host-encoded, normal prion protein called PrP^C^ [2]. PrP^C^ is a cell-surface localized glycoprotein that is widely expressed in a variety of tissues and reaches a very high level in the central nervous system (CNS) [5,6]. During prion disease, a portion of the PrP^C^ molecules convert to PrP^Sc^, which is aggregated and resistant to protease digestion [2]. The most important pathogenic feature of PrP^Sc^ is its ability to seed the conversion of naïve PrP^C^ into PrP^Sc^, which forms the basis for prion infectivity. The resulting PrP^Sc^ accumulation in diseased individuals or animals leads to neurodegeneration and clinical manifestations of the disease. 

It is now well accepted that there are a wide variety of prion strains and each strain causes disease with a distinct incubation time and neuropathology [7,8,9,10]. Small structural differences among various PrP^Sc^ aggregates are believed to be the molecular basis for different prion strains [11]. In the same host, prion strains are highly stable. But when a prion strain is passaged to a new host, its properties may change. The difficulty in transmitting a stabilized prion strain to a different host is called a transmission barrier [9]. The adaptation of a prion strain to the new host, i.e., acquiring new biochemical and/or pathogenic properties, is known as strain mutation or evolution [9,12]. 

Many efforts have been made to develop therapies against prion disease [13,14]. Because of the central role of PrP^Sc^-seeded PrP conversion in pathogenesis, there are clear therapeutic targets: (1) the pathogenic PrP^Sc^; (2) the normal PrP^C^; and (3) the downstream cellular events that leads to neurotoxicity. Many approaches have been explored against these targets, including small molecules, vaccination, antibodies, peptide aptamer, and various nucleic acid-based agents [13,14,15,16,17,18]. Some of the recent attempts, such as the anti-PrP antisense oligonucleotides [19] and cellulose ethers [20,21,22], have shown great prophylactic and therapeutic potentials. The most advanced therapeutic development is PRN100, a humanized anti-PrP^C^ monoclonal antibody ICSM18, which has a demonstrated efficacy in extending the survival of prion-infected mice [23] and is currently undergoing human trials [24]. In this review, we focus our discussions on the immunological approaches against prion disease. 

## 2. Why Is Immunotherapy a Plausible Choice for Prion Disease?

Immunotherapy, known for its strong specificity and relatively few side effects, is considered a promising strategy for many now-incurable human diseases, including neurodegenerative disorders [25,26]. Enormous efforts have been spent to develop immunotherapies against Alzheimer or Parkinson disease [27,28,29,30]. Despite disappointing outcomes from several clinical trials and the potential adverse effect of amyloid-related imaging abnormalities, the reanalysis of clinical trial data of Aducanumab, a human antibody targeting Aβ aggregates, revealed a consistent improvement in treated-patients, supporting that immunotherapy should still be considered a viable therapeutic approach for Alzheimer’s disease [31]. Compared with other late age onset neurodegenerative diseases, prion disease has unique advantages for immunotherapy. First, the misfolded PrP^Sc^ form is a well-established disease-causing agent [2,32,33,34], which makes it a valid therapeutic target. Second, PrP is a cell-surface localized protein and the PrP^C^-to-PrP^Sc^ conversion occurs on the cell surface or along the endocytic pathway [2,5], which is easily accessible to therapeutic agents.

Ideally, therapies should target the disease-causing PrP^Sc^, which avoids the potential toxic effects by binding to certain regions of PrP^C^ [35] or by targeting cellular processes that are important for the normal physiological functions [36,37]. Many efforts have been made to search for small molecules targeting PrP^Sc^ and some of them appeared to be quite promising [38]. However, a problem for the small-molecule approach is the presence of minor structural differences among various prion strains or prions from different species [9]. It is well known that a difference of a few amino acids, sometimes just one or two, is sufficient to create a strong barrier to prion transmission [9], indicating that PrP^Sc^ from different prion strains (or species) must differ in the structure around those amino acids. In other words, the structural differences among different prion strains or prions from different species are likely in the regions that are crucial for its seeding activity, which explains the existence of the transmission barrier and the faithful propagation of the unique prion conformation of each strain. Small molecules target a small region of a protein, and to be effective, these molecules would need to target a region that is crucial for the seeding activity. Thus, a small molecule that fits the seeding region is likely to be a good inhibitor for a given prion strain, but the likelihood for such a small molecule to have a universal anti-prion effect is not going to be high. Indeed, carefully developed small molecules (e.g., IND24) showed a very promising efficacy against murine prions but were less (or not at all) effective against sheep or human prions [13,39,40].

Despite the minor structural differences among prion strains, a consensus in the field is that all PrP^Sc^ molecules do share a common overall architecture. This view is consistent with the high homology among all mammalian PrPs, the high-β-sheet content in all PrP^Sc^, the strong C-terminal PK resistance of almost all PrP^Sc^ molecules, and the findings from structural studies of PrP^Sc^ [1,2,41,42,43]. Therefore, a good therapeutic molecule with a wide-spectrum anti-prion effect would need two properties: (1) the ability to interact with a relatively large area of PrP^Sc^ and (2) a tolerance of small structural variations among different prion strains. 

Relative to small molecules, antibodies appear to be a better choice for prion disease therapeutics. Antibodies bind a linear epitope of at least several amino acids and can usually tolerate a difference of 1 or 2 amino acids [44]. If the epitope is conformational, the antibody–antigen contacts will be even wider [44,45]. Therefore, the antibody–PrP^Sc^ interaction will be more tolerant of small structural variations, increasing the likelihood of recognizing the shared overall PrP^Sc^ structure. This is particularly true for a vaccine-based approach, which will elicit a polyclonal response that, at least theoretically, will target multiple epitopes on the surface of PrP^Sc^. In this case, minor structural variations are unlikely to affect the efficacy. 

The public health concern about CWD is another reason to consider immunotherapy [46]. As of November 2019, CWD in free-ranging deer, elk, and moose has been found in at least 24 states in the United States and three provinces in Canada [47]. CWD has also been reported in reindeer and moose in Norway, Finland, and Sweden [48], as well as in South Korea following the importation of CWD-infected elk [49,50]. The incidence of CWD in free-ranging deer and elk is relatively low, but the prevalence may exceed 30% in endemic areas and can be as high as 80–90% in a captive herd [47,51,52]. So far, there is no evidence that CWD can be transmitted to humans [52,53]. However, CWD is able to transmit to a variety of other animal species, including nonhuman primates [54,55]. The uncontrollable spread of CWD in free-ranging animals, the ability to transmit the disease to other animal species in close contact to CWD-infected carcass or body fluids, and the fact that a prion can evolve when it is exposed to a new environment [56], raise the concern that the CWD prion could evolve into an agent that is harmful to human health [46]. 

Immunotherapy, especially active immunization with appropriate vaccines, is probably the most practical approach to combating CWD. Although antibodies are unable to cross the blood–brain barrier to alter neurodegeneration in the CNS, vaccination is still an effective measure for controlling CWD. In diseased animals, the antibodies induced by vaccination will at least reduce PrP^Sc^ in the peripheral tissues and prevent the shedding of CWD prions, which is believed to be a main reason for the efficient lateral transmission of CWD [57,58]. In uninfected animals, the presence of anti-PrP antibody in the peripheral tissues would prevent the infection via peripheral route, which is the natural route for CWD prion transmission [59,60]. 

These reasons support that immunotherapy is a plausible and practical therapeutic strategy against human and animal prion diseases. Because of the lower incidence, passive instead of active immunization is probably more appropriate for human prion disease. For animal prion disease, particularly the highly contagious diseases like CWD, developing an effective active immunization approach would be the most effective strategy. Over the past two decades, prion researchers have studied both active and passive immunological approaches against prion disease. Results of these studies are reviewed and summarized in the next two sections. 

## 3. Active Immunization against Prion Diseases

Eliciting an immune response against PrP in wild-type animals faces the challenge of self-tolerance. PrP is widely expressed and its expression in a variety of tissues can induce strong self-tolerance [61], which is likely due to the exposure of native PrP for clonal deletion of T and B cells. Certain epitopes on bacterially expressed recombinant PrP or synthetic PrP peptides are able to induce immune responses in wild-type animals, but the elicited antibodies do not recognize cell surface localized native PrP [61,62]. These findings indicate that some epitopes are masked on native PrP, possibly due to post-translational modifications or folding of native PrP, or its interaction with other molecules. These epitopes are available on recombinant PrP, synthetic PrP peptides, or denatured PrP, but the elicited immune responses are not useful because the antibodies do not bind native PrP or prevent prion propagation. Thus, for active immunization, it is important to differentiate whether the elicited immune response is because of the exposure of masked epitopes on native PrP or a true break of self-tolerance to native PrP.

Over the years, many groups have explored modified PrPs as immunogens to break such tolerance, including truncated or modified PrP peptides [63,64,65,66,67], PrP dimers [68,69], heterologous PrP peptides [70], and crosslinked PrP peptides [71]. Moreover, certain types of adjuvants, such as CpG-oligodeoxynucleotides (CpG), have been reported to help break self-tolerance [66]. These studies are discussed below, but note that in almost all these studies, if there is any effect on the survival of prion-infected mice, the effect is generally marginal. Further improvements are critically needed to enhance the efficacy of active immunization against prion disease. 

The PrP131–150 and PrP211–230 peptides contain MHC-II binding motifs, and when these peptides were used as immunogens, strong immune responses were detected in wild-type mice [63]. In mice transplanted with prion-infected N2a cells, this vaccination strategy was able to reduce the protease-resistant PrP^Sc^ [63]. Bone-marrow-derived dendritic cells (DCs) loaded with PrP98–127 or PrP158–187 peptide also overcame immune tolerance and prolonged the lifespan of mice infected with 139A strain of murine prion [67]. Another commonly used strategy is to conjugate PrP or PrP peptides to molecules that stimulate immune response. Conjugating bacterial Hsp70 homologue DnaK to PrP23–230 induced anti-PrP antibody in BALB/c mice [72]. Keyhole limpet hemocyanin (KLH) linked to PrP105–125 peptide prolonged the survival times of mice orally infected with prion [73]. PrP^Sc^-absorbed Dynabeads appeared to induce a PrP-specific IgM response and extended the survival time of FVB/N mice that received intraperitoneal inoculation of RML prion [74]. Covalently linked recombinant PrP dimer in C57BL/6 mice effectively induced polyclonal anti-PrP antibodies that were able to reduce the amount of PrP^Sc^ in prion-infected cells [75]. More recently, dimeric deer PrP was shown to successfully induce anti-PrP antibody in transgenic mice overexpressing deer PrP, and the post-immune serum inhibited prion seeding activity in vitro [68]. Combining the use of CpG adjuvant and recombinant deer or mouse PrP monomer or dimer as the immunogen, Abdelaziz et al. recently demonstrated that the vaccination strategy prolonged the survival of elk PrP expressing transgenic mice exposed to CWD prion peripherally [69]. They also showed that the same strategy was able to break the self-tolerance in reindeer, a natural host of CWD. 

An alternative approach to break self-tolerance is to use heterologous recombinant PrPs as immunogens. It has been shown that bovine (and to a less extent, sheep) recombinant PrPs induced anti-PrP antibody production and increased the incubation times of Fukuoka-1 prion-infected mice [70]. Moreover, Ishibashi et al. discovered that the bacterial succinylarginine dihydrolase (SADH) contained an amino acid sequence similar to the epitope recognized by the 6H4 anti-PrP monoclonal antibody. Using recombinant SADH as an immunogen, they showed that this strategy is able to extend the survival of prion-infected mice [76]. 

DNA vaccines are known to enhance an immune response. PrP-specific IgG in the serum has been successfully induced by either (1) immunization with cDNA encoding for heterologous PrP fused to a targeting protein that enhances antigen uptake and presentation via major histocompatibility complex class I [77] or (2) a stimulatory T-cell epitope peptide [78], but whether the DNA vaccines could prevent prion infection was not determined. 

Using an attenuated Salmonella vaccine strain expressing PrP, Goni et al. showed that mucosal vaccination overcomes the immune tolerance and induces both intestinal anti-PrP IgA and systemic anti-PrP IgG, which results in a significant delay of prion disease in mice orally infected with 139A prion [79,80]. A similar strategy is also able to extend the survival of deer orally challenged with CWD-infected brain homogenates, particularly in animals with higher titers of anti-PrP antibodies [59]. 

The active immunization strategies are generally directed against the normal host-encoded prion protein, PrP^C^, and the protective effect against prion disease is presumably due to the stabilization of PrP^C^ and thereby preventing the PrP^C^-to-PrP^Sc^ conversion. However, as mentioned above, the ideal target would be PrP^Sc^. Because PrP^Sc^ is structurally distinct from PrP^C^, it is possible that the immune system may recognize PrP^Sc^ as a foreign invader and elicit an immune response. It has been reported that some anti-PrP antibodies can be detected towards the end stage of prion disease [59,81], suggesting that there is some type of immune response against the aberrantly folded PrP molecules. The attempts to induce PrP^Sc^-specific immune responses have been mainly focused on epitopes that are believed to be uniquely exposed in PrP^Sc^. The YYR motif, a YML motif in β-sheet 1, and the rigid loop linking β-sheet 2 to α-helix 2 were explored as vaccines and they did induce sustained PrP^Sc^-specific antibody responses [82,83]. Using a nonreplicating human adenovirus expressing the rigid loop epitope fused with rabies glycoprotein G, Taschuk et al. showed that oral immunization of this vaccine in white-tail deer is able to induce both systemic and mucosal anti-PrP antibodies, but whether this vaccination approach is protective against prion disease remains unknown [84]. The effect of YYR vaccine on a native host was tested in elk that were housed in a prion contaminated environment and vaccinated with YYR fused with a carrier protein, Leukotoxin from *Mannheimia haemolytica* [85]. Although the vaccination induced YYR-specific antibody, it paradoxically accelerated the onset of disease in elk with the 132MM genotype and showed no effect in elk with the 132ML genotype. Because of the limited study groups due to the usage of a large animal, it is difficult to pin down the reason for the acceleration of the disease. 

Overall, studies of active immunization clearly show that multiple strategies are able to break self-tolerance and induce anti-PrP immune responses, but the therapeutic or preventive effect on prion-infected animals is generally not optimal (Summarized in Table 1). 

## 4. Passive Immunization against Prion Diseases

Because of the availability of PrP knockout mice and the hope to generate PrP^Sc^-specific antibodies, a lot of anti-PrP antibodies have been generated, but so far, none of them have demonstrated specificity for PrP^Sc^. Based on the idea that antibody binding to PrP^C^ may prevent the PrP^C^-to-PrP^Sc^ conversion, the potential of these PrP^C^-binding antibodies against prion replication and prion disease has been extensively studied. 

The ability of antibodies to inhibit PrP conversion has been mainly evaluated in vitro using prion-infected cultured cells [96,97,98,99]. The 6H4 antibody (directed at residues 144–152) eliminated PrP^Sc^ in prion-infected neuroblastoma (N2a) cells [96]. PrP-specific Fab fragments D13 (recognizing residues 95–103) and D18 (recognizing residues 132–156) effectively clear PrP^Sc^ from prion-infected N2a cells [97]. Two studies screened two large panels of anti-PrP antibodies and identified several that were capable of reducing PrP^Sc^ in prion-infected N2a cells [99,100]. Notably, the effective antibodies recognize a variety of epitopes in different regions of PrP, indicating that the observed inhibitory effects may work through different mechanisms. 

The efficacy of anti-PrP antibodies against prion disease was determined using prion-infected mice (Summarized in Table 2). It was shown that weekly i.p. injection of 8B4 (recognizing residues 34–52) or 8H4 (recognizing residues 175–185) anti-PrP antibody extended the survival time of CD-1 mice that had received i.p. inoculation of 139A prion [101]. The most impressive therapeutic effect was achieved with ICSM 18 (recognizing residues 146–159) or ICSM 35 (recognizing residues 91 to 110) [23]. When 2 mg of antibody was given i.p. twice weekly, mice that had received i.p. RML prion inoculation survived more than 500 days with no sign of prion disease. However, the treatment was not effective when the antibody treatment was started at later stages of prion infection (at clinical onset) or when mice were infected via intracerebral prion inoculation, indicating that these antibodies are unable to cross the blood–brain barrier to stop disease progression in the CNS. 

The effect of a short antibody treatment was also tested. Using 6D11 anti-PrP antibody, Sadowski et al. showed that a 4-week or 8-week treatment of i.p. antibody injection was able to extend the survival of mice infected with 22L prion via i.p. route [102]. To determine the effect of antibody treatment at late stages of prion infection, Song et al. performed an intraventricular infusion of anti-PrP antibodies [103]. When the treatment started at 60, 90, or 120 days after prion infection, a 4-week infusion prolonged survival time for a short period of time. Although the effect is small, this result showed that anti-PrP antibody in the CNS is able to alter prion disease progression.

## 5. Difficulties and Potential Approaches to a Successful Immunotherapy against Prion Disease

Significant progress has been made in immunological approaches against prion disease. Yet, the development of an effective immunological strategy still faces a number of obstacles [108], including the safety of the treatment, the blood–brain barrier, and the difficulties in developing a PrP^Sc^-targeted therapy.

Safety—PrP^C^ is a host-encoded protein and CNS delivery of several anti-PrP antibodies has been shown to cause neurotoxicity [35,109,110,111]. It was first reported that stereotactic injection of monoclonal antibody D13 or IgG P (recognizing epitopes within residues 95–105), but not their monovalent Fab fragments, caused acute neurotoxicity in the brain [109]. Lefebvre-Roque et al. showed that intraventricular infusion of 4H11 antibody, or its bivalent or monovalent Fab fragments caused neuronal loss and gliosis [111]. But Klöhn et al. reported that stereotactic injection of 2 mg of ICSM35 or ICSM18 antibody, or previously reported toxic D13 or IgG P antibody failed to induce acute neuronal apoptosis [112]. The same antibodies were tested in a separate study and found to cause neurotoxicity at higher dosages [35]. Sonati et al. performed a comprehensive study of a large group of anti-PrP antibodies and reported that 8 out of 12 antibodies recognizing the folded globular domain of PrP (residues 124–230) caused acute neurotoxicity, whereas 3 high affinity antibodies recognizing the octarepeat region (residues 50–90) did not [110]. Divalent or monovalent Fab fragments or recombinant single-chain variable fragments are as toxic as the antibodies and the POM1 antibody appeared to be the most toxic. Interestingly, pre-incubation of the non-toxic octarepeat-recognizing antibodies eliminated POM-1-induced toxicity [110]. The similarities between the neurotoxicity caused by the globular domain-binding antibodies and prion infection [113] suggest that the octarepeat-binding antibodies have a great therapeutic potential. This view is consistent with a recent report showing that the octarepeat-binding human Fabs prevent neurotoxicity caused by prion infection [114]. In the same study, Senatore et al. also identified a small number of human carriers of high-titer anti-PrP antibody and found no specific pathologies associated with the presence of these antibodies. This finding is highly intriguing, which suggests that self-tolerance to PrP can be broken and certain anti-PrP antibodies appear to be innocuous in human. Altogether, the anti-PrP antibody-induced neurotoxicity appears to be epitope- and dosage-dependent [35]. The potential neurotoxic effects of immunological approaches against prion disease must be carefully evaluated. 

For passive immunotherapy, each therapeutic antibody needs to be carefully characterized to prevent the potential adverse effect. The active immunization, however, induces polyclonal antibody response and some of those antibodies may target the neurotoxic epitopes of PrP^C^. One way to overcome this obstacle is to select immunogens that are conformationally different from PrP^C^ but similar to the pathogenic PrP^Sc^. Because of the conformational difference between these immunogens and PrP^C^, and the self-tolerance of the host to native PrP^C^, this approach may direct the immune response more toward PrP^Sc^ and avoid neurotoxicity. The studies of recombinant PrP aggregates in vitro have generated a series of potential immunogens, such as recombinant PrP amyloid fibrils [115], multimeric recombinant PrPs [68], and the nonpathogenic, PK-resistant recPrP^R-low^ aggregates that share many structural features of pathogenic PrP^Sc^ [116,117]. These PrP forms should be tested as immunogens in future studies. 

Another caveat that should be considered is the choice of adjuvant for active immunization. It was reported that repeated CpG administration severely damages peripheral lymphoid tissues [118]. Because peripheral prion infection requires prion replication in follicle dendritic cells [119], a careful analysis of lymphoid tissues is required to determine whether the anti-prion effect is due to the tissue damage or to a real anti-prion effect. This complication can be mitigated by using a lower dosage or choosing other adjuvants, which have been used in recent studies [68,69].

Blood–brain barrier—The size of a conventional antibody is approximately 150 kDa, which makes it difficult to cross the blood–brain barrier [120]. This is not a major problem for using immunization as a preventive measure because peripheral prion infection is the natural transmission route, but it is a significant problem for using an antibody as a therapy. As demonstrated by Song et al., antibodies administered i.p. eliminated disease caused by peripheral prion infection but had no effect on disease caused by intracerebral prion inoculation [103]. Intrathecal antibody administration appears to be an easy solution, but the need for a large dose of anti-PrP^C^ antibody in treating prion disease would require repeated intrathecal administrations, which may not be ideal. Several technologies have been developed for CNS delivery, including receptor-mediated transport, viral vector-based gene delivery, or nanomedicines combined with antibody engineering [121,122,123,124]. Notably, brain delivery of a single-chain fragment of antibody D18 (scFvD18) using an AAV2 or AAV9 vector prolonged the survival time of prion-infected mice and decreased the amount of PrP^Sc^ in the brain [105,107], which was consistent with a previous study in transgenic mice [104]. In addition, it is known that the blood–brain barrier is weakened during neurodegeneration [125], but how permeable it is during human prion disease is unknown. If antibodies are able to enter CNS during prion disease, both peripheral antibody administration and therapeutic vaccines can be considered. 

Anti-PrP^Sc^ antibody—Compared with the anti-PrP^C^ antibodies, a PrP^Sc^-specific antibody would be a better choice for developing a successful immunotherapy. Yet, despite extensive efforts, there is still no confirmed antibodies that specifically target PrP^Sc^ and do not react with PrP^C^. The advance in structural studies of the pathogenic PrP^Sc^ [126,127,128,129] may lead to the development of such an antibody. In addition, a nanobody, the antigen-binding region of the camelid single-chain antibody [130], could be an alternative approach. A nanobody is small (15 kDa) and has a long, protruding CDR3 that prefers conformational epitopes. Since the difference between PrP^C^ and PrP^Sc^ is conformational, nanobodies are ideal candidates to be a PrP^Sc^-specific agent. The fact that nanobodies are small and encoded by a single gene also allow them to be easily modified and packaged into viral vectors for gene therapy. Several PrP^C^-binding nanobodies have been identified, and some of them are able to inhibit prion replication in vitro or in prion-infected cell lines and to cross the blood–brain barrier [45,131,132,133]. However, the in vivo anti-prion effect of these nanobodies remains to be determined and thus far, no PrP^Sc^-specific nanobodies have been identified.

## 6. Conclusions

Promising results have been achieved with both passive and active immunization strategies against prion diseases. We envision that advances in the following areas will bring us closer to a successful immunotherapy against prion disease. First, the potential of various PrP^Sc^-like, noninfectious, recombinant PrP conformers as vaccines should be tested to determine whether they are able to elicit an immune response more toward PrP^Sc^ and to stop the pathogenic changes. Second, the availability of new technologies will allow the search for a PrP^Sc^-specific antibody to be more feasible. If successful, it will be a safer and more disease-targeted therapeutic agent. Third, new methods need to be developed to efficiently deliver the therapeutic antibodies to CNS. Given the rapid progression of prion disease after clinical onset and the accumulation of PrP^Sc^ in the brain at the clinical stage, these delivery methods will be crucial for an effective therapy.

Although it is not the focus of this review, it is important to point out that a large body of recent literature indicate the involvement of PrP in the pathogenesis of other common neurodegenerative diseases such as Alzheimer’s and Parkinson’s diseases [134,135,136,137,138,139,140]. Therefore, passive immunization of anti-PrP antibodies may have a broader application that is beyond prion disease. 

## Figures and Tables

**Table 1:** Active immunization strategies against prion diseases.

**Immunogen**		**Adjuvant**	**Administration Route**	**Prion Inoculation**	**Outcome**	**Reference**
Monomeric PrP	Mouse recombinant PrP treated with urea	Freund’s adjuvant	Subcutaneous	Intraperitoneal inoculation with 139A prion in CD-1 mice	The survival time was prolonged by 9.25% (189 ± 4/173 ± 2 days)*.	[86]
	Bovine PrP25–242	Freund’s adjuvant	Intraperitoneal	Intraperitoneal inoculation with Fukuoka-1 prion in BALB/c mice	The survival time was prolonged by 10.65% (322 ± 15/291 ± 10 days)*.	[70]
	Mouse recombinant PrP cross-linked with DnaK	Freund’s adjuvant	Intraperitoneal	N/A	Antibodies against PrP were detected.	[72]
Multimeric PrP	Mouse recombinant PrP tandem dimer	Freund’s adjuvant/CpG/TiterMax	Subcutaneous	N/A	Anti-PrP antibodies were induced, which suppressed PrP^Sc^ propagation in cultured cells.	[75]
	Mouse recombinant PrP tandem dimer	CpG	Subcutaneous	N/A	Self-tolerance was broken; CD4 and CD8 T cell responses were detected.	[87]
	Mouse recombinant PrP tandem dimer	Freund’s adjuvant	Subcutaneous	Intraperitoneal inoculation with RML prion in mice	Anti-recombinant PrP antibodies were induced when the immunization was coupled with the administration of anti-OX40 antibody; The induced antibodies do not recognize native PrP in mice and had little effect in delaying prion pathogenesis.	[61]
	Deer PrP dimer/deer PrP monomer/mouse PrP dimer/mouse PrP monomer	CpG	Subcutaneous	Intraperitoneal inoculation of CWD prion in transgenic mice expressing elk PrP	Survival time was prolonged by 24.13%, 28.40%, 15.94% or 59.93% (142.5 ± 5.8, 147.4 ± 13.4, 133.1 ± 15 or 183.6 ± 8.8/114.8 ± 10 days)*.	[69]
	Multimeric recombinant cervid PrP	CpG	Subcutaneous	N/A	Auto-antibodies were induced, which interfere with in vitro prion conversion.	[68]
	Aggregated PrP	Freund’s adjuvant	Subcutaneous	Intraperitoneal inoculation of RML prion in mice	The survival time was prolonged approximately by 14% (228/200 days)*.	[88]
PrP fragment or peptide	Mouse recombinant PrP90–231	CT (cholera toxin)	Intranasal	Oral inoculation with 139A prion in BALB/c mice	The median survival time was prolonged by 3.30% (266.0/257.5 days)*.	[89]
	Mouse PrP131–150/PrP211–230	Freund’s adjuvant	Hind footpad injection	N/A	The peptides were strongly immunogenic in both NOD and in C57BL/6 mice.	[63]
	Mouse PrP98–127/PrP158–187	CpG + Freund’s adjuvant	Subcutaneous	Intraperitoneal inoculation of 139A prion in C57 BL/6 mice	The mean survival time was prolonged by 8.10% or 5.71% (227 ± 8 or 222 ± 14/21 0± 8 days)*.	[90]
	Mouse PrP141–159/PrP165–178 conjugated to BCP	Adjuvac^TM^	Intramuscular	Intraperitoneal inoculation of RML prion in C57 BL/6 mice	The mean survival time was prolonged by 8.41% or 6.54% (232 ± 12 or 228 ± 19/214 ± 8 days)*.	[91]
	Mouse PrP105–125 linked to KLH	Montanide IMS-1313	Intraperitoneal	Oral inoculation with 139A prion in NMRI mice	The survival time was prolonged by 11.22% (223 ± 18/200.5 ± 10 days)*.	[73]
	Hamster PrP105–128/119–146/142–179 conjugated to mcKLH	Freund’s adjuvant	Intramuscular, subcutaneous, and intradermic	Intraperitoneal inoculation with 263K prion in hamster	Average survival time was prolonged by 12.95%, 18.71% or 18.71% (157 ± 49, 165 ± 43 or 165 ± 54/139 ± 24 days)*, but without significant statistical differences.	[71]
PrP-loaded DCs	Mouse PrP98–127 loaded dendritic cells (DCs)	N/A	Intraperitoneal	Intraperitoneal inoculation of 139A prion in C57 BL/6 mice	The median total survival time was extended by 18.69% (254/214 days)*.	[67]
	Human recombinant PrP (encoded by adenovirus) loaded dendritic cells (DCs)	N/A	Intramuscular	Intraperitoneal inoculation of 139A prion in C57 BL/6 mice	Compared with prion infected only group, survival times were prolonged by more than 7% in all vaccinated groups, including the DC control groups.	[92]
Oral vaccine using a Salmonella vector	Mouse PrP-TetC expressed by Salmonella	Alum	Oral	Oral infection with 139A prion in CD-1 mice	30% of each treatment group were alive and without clinical signs of infection by 500 days (control groups showed clinical signs of prion infection by 300 days).	[79]
	Mouse PrP-TetC expressed by Salmonella	Alum	Oral	Oral inoculation of 139A prion in CD-1 mice	At 400 days post-inoculation, 100% of the animals in the high IgG, high IgA group (n = 14) were free from clinical symptoms, (control groups had shown clinical signs of prion infection by 205 days).	[80]
	Mouse/cervid PrP expressed by Salmonella	Alum	Oral	Oral inoculation of CWD prion in white-tailed deer	The median survival time was prolonged by 51% (909/602 days)*.	[59]
Antigen mimicry	Recombinant succinylarginine dihydrolase E/S (SADH-E/S)	Freund’s adjuvant	Intraperitoneal	Intraperitoneal inoculation of Fukuoka-1 in BALB/c mice	Anti-PrP auto-antibodies with anti-prion activity were induced and the survival time of prion infected mice was prolonged by 10.40% or 7.72% (329 ± 15 or 321 ± 15/298 ± 28 days)*.	[76]
DNA vaccine	Plasmid pcDNA3.1-PrP/-Ubiq-PrP/-PrP-LII/-PrP-ER (human)	N/A	Intraperitoneal	N/A	Vaccination with PrP DNA followed by protein boosting induces PrP-specific PrP-specific antibody response and T-cell mediated responses.	[77]
	Plasmid pcDNA3.1-PrP(human)	Freund’s adjuvant	Tibialis anterior muscles injection	N/A	The antibodies against the native form of human PrP and autoantibodies against the native form of murine PrP^C^ were generated.	[78]
	Plasmid pCMV–UbPrP/pCMV–PrPLII (mouse)	N/A	Anterior tibial muscle	Intracerebral inoculation of BSE prion in 129/Ola mice	Average survival time was prolonged by 10% (22/20 weeks)*.	[93]
	Plasmid pCG-PrP-P30 (mouse)	CpG	Intradermal and subcutaneous	Intraperitoneal inoculation with RML prion in C57 BL/6 mice	Failed to protect wild-type mice from prion infection.	[94]
PrP^Sc^-specific epitopes	ICSM35-Dynabeads-RML/ICSM18-Dynabeads-RML	Freund’s adjuvant	Intraperitoneal	Intraperitoneal inoculation of RML prion in FVB/N mice	The mean survival time was prolonged by 10.89% (224 ± 16 or 224 ± 22/202 ± 2 days)*.	[74]
	Epitope (QVYYRPVDQYSNQN)-Lkt fusion protein	Emulsigen-D	Subcutaneous	N/A	PrP^Sc^-specific IgG antibody responses were induced following two vaccinations.	[95]
	Ad5: tgG-RL (RL epitope)	N/A	Oral	N/A	PrP^Sc^-specific systemic and mucosal antibody responses were induced.	[84]
	Epitope (QVYYRPVDQYSNQN)-Lkt fusion protein	Emulsigen-D	Intramuscular	Elk in CWD contaminated pens	Accelerated onset and PrP genotype dependent shortening of survival time in vaccinated group were observed.	[85]

* Survival time of vaccinated group/survival time of control group.

**Table 2:** Passive immunization strategies against prion diseases.

Antibody	Properties of Antibody	Administration Route	Prion Inoculation	Outcome	Reference
6H4μ	IgG1 monoclonal antibody recognizing residues 144–152 of murine PrP^C^	Transgene	Intraperitoneal inoculation with RML prion in transgenic mice.	The i.p. prion inoculation induced pathogenic changes were prevented in 6H4μ transgenic mice, but not in 15B3μ transgenic mice.	[104]
15B3μ	IgM antibody recognizing PrP^Sc^-like PrP aggregates				
ICSM35	IgG2b monoclonal antibody with high affinity for both murine PrP^C^ and PrP^Sc^, recognizing residues 91–110 of murine PrP	Intraperitoneal injection started 7 days or 30 days post prion inoculation; 2 mg antibody was injected twice weekly.	Intraperitoneal inoculation with RML prion in FVB/N mice.	Animals remained healthy for over 300 days after equivalent untreated animals had succumbed to the disease.	[23]
ICSM18	IgG1 monoclonal antibody recognizes residues 146–159 of murine PrP with a lower affinity for PrP^Sc^				
8B4	Monoclonal antibody recognizing residues 34–52 of PrP	Intraperitoneal injection started immediately after prion infection; 50 µg of antibody was injected weekly after.	Intraperitoneal inoculation of 139A prion in mice.	The onset of clinical symptoms was delayed.	[101]
8H4	Monoclonal antibody recognizing residues 175–185 of PrP				
8F9	Monoclonal antibody recognizing residues 205–233 of PrP				
106	IgG2b monoclonal antibody recognizing residues 88–90 of murine PrP	Intraventricular infusion	Intracerebral inoculation of Obihiro or Chandler prion	Antibody 31C6 treatment prolonged the survival time about 8% even when the treatment started at very late stage of prion disease.	[103]
110	IgG2b monoclonal antibody recognizing residues 83–89 of murine PrP				
31C6	IgG1 monoclonal antibody recognizing residues 143–149 of murine PrP				
44B1	IgG2a monoclonal antibody recognizing a discontinuous epitope within residues 155–231 of murine PrP				
scFvs	AAV2 delivery of single chain variable fragment of D18 antibody (scFvD18) recognizing residues 132–156 of PrP, or anti-PrP scFvs (scFv3:3, scFv6:4, and scFv6:6) obtained by screening a human scFv library.	Intracerebral delivery	Intraperitoneal inoculation of RML prion 1 month after the AAV2 delivery.	Treatment with scFvD18 or scFv3:3 extended mean incubation time by 25.63% or 11.56%.	[105]
6D11	Monoclonal antibody recognizing residues 97–100 of murine PrP	Intravenous injection started with 1 mg of antibody immediately after prion infection; 0.5 mg antibody was injected intraperitoneally twice weekly for 4 or 8 weeks.	Intraperitoneal inoculation of 22L prion in CD-1 mice.	Antibody treatment prolonged the incubation period by 36.9% and ameliorated CNS pathology.	[102]
W226	Monoclonal antibody recognizes residues 146–159 of murine PrP	Intraperitoneal injection started 28 days post prion inoculation; 2 mg of antibody was injected twice weekly.	Intraperitoneal inoculation of RML prion	Incubation period of prion infected mice was slightly increased.	[106]
scFvD18	AAV9 delivery of single chain variable fragment D18 (scFvD18) recognizing residues 132–156 of PrP	Intracerebral delivery	Intraperitoneal inoculation of RML prion one month after the delivery of AAV9-scFvD18.	Incubation time was slightly increased and the amount of PrP^Sc^ in the brain was reduced.	[107]
